# Property Characterization and Mechanism Analysis of Polyoxometalates-Functionalized PVDF Membranes by Electrochemical Impedance Spectroscopy

**DOI:** 10.3390/membranes10090214

**Published:** 2020-08-30

**Authors:** Lei Yao, Ziyi Long, Zhe Chen, Qisong Cheng, Yuan Liao, Miao Tian

**Affiliations:** 1 School of Electrical and Information Engineering, Wuhan Institute of Technology, Wuhan 430205, China; lzy950410@163.com (Z.L.); cqs1129@163.com (Q.C.); 2 Hubei Key Laboratory of Plasma Chemical and Advanced Materials & School of Materials Science and Engineering, Wuhan Institute of Technology, Wuhan 430205, China; zrickchen@163.com; 3 Sino-Canadian Joint R&D Center for Water and Environmental Safety, College of Environmental Science and Engineering, Nankai University, Tianjin 300350, China; liaoyuan@nankai.edu.cn; 4 School of Ecology and Environment, Northwestern Polytechnical University, 1 Dongxiang Road, Chang’an District, Xi’an 710129, China

**Keywords:** polyoxometalates, membrane, electrochemical impedance spectroscopy, PVDF, APTMS

## Abstract

Polyoxometalates (POMs) has proved its advantage in constructing high-performance nanocomposite membranes such as catalytic membranes, adsorptive membranes, and forward osmosis membranes. However, it is challenging or tedious to characterize its distribution and effect on the membrane structures due to the equipment resolution limitation, discrete nano-scaled structures of POMs, and limited doping amount compared to the polymeric membrane matrix. In this paper, POMs-functionalized polyvinylidene fluoride (PVDF) membranes were fabricated by phase inversion combined with the sol-gel method, and electrochemical impedance spectroscopy (EIS) was utilized to analyze the nanocomposite membrane intrinsic properties. Through adjusting the additives in the sol-forming process, a set of membranes with varied intrinsic properties were developed accordingly. The wetting degree of the membranes related to the hydrophilic nature of the membrane surfaces had a crucial influence on the impedance measurements at the early stage. Through EIS analysis, it was demonstrated that the amination of the membrane matrix through (3-aminopropyl)trimethoxysilane (APTMS) treatment and the immobilization of POMs through electrostatic attraction would not generate new pore structures into the membrane and only alter the membrane surface roughness and composition. To my knowledge, it is the first time that EIS was utilized to characterize the hydrophilicity of the membranes and pore structures of the POMs-modified membranes. Our findings indicate that EIS can provide valuable information for probing the structures of other nano-materials-incorporated membranes.

## 1. Introduction

Membrane technology has been demonstrated to be efficient for water treatment over the past decades. Inspired by the multi-functional characteristics of biomimic membranes, incorporation of nanomaterials, namely nanocomposite membranes, has been a promising approach to tailor the membrane structure and impart additional unique properties in the past decade [1]. Up to date, an extensive range of nanomaterials have been incorporated into membranes, such as carbon nanotube (CNT) [2], graphene oxide (GO) [3], metal-organic frameworks (MOF) [4], polyoxometalates (POMs) [5,6,7], etc. [8], to enhance membrane separation efficiency or other properties. The characterization of their distribution and effect on the membrane structures is crucial and will shed light on nanocomposite membrane development. In general, scanning electron microscopy (SEM), atomic force microscopy (AFM), as well as transmission electron microscopy (TEM) are frequently utilized to directly observe the pore structures, distribution of nanomaterials, and roughness of the membranes, respectively. These techniques are effective for the observation of nanomaterials with an obvious structural contrast with the polymeric matrix. When the incorporated amounts of nanomaterials were controlled to be moderately low (~0.1%) given the cost and dispersibility considerations, or their distribution is changeable in applications, the above-mentioned characterization methods became impotent. For instance, by constructing a GO-selective layer on the membrane surface, precise ionic and molecular sieving in aqueous solution with high-flux could be obtained [3]. However, the interlayer spacing of GO membranes is highly related to the ionic aqueous environment and changeable under different concentrations of feed solution. Therefore, the observation of GO membrane morphology by SEM, TEM, and AFM, which are typically operated in a vacuum environment, can hardly establish the correlation between the membrane structure and membrane performances. Other techniques, for example, electrochemical impedance microscopy (EIS) [9], has showed its advantage in providing more information of the membrane structures in such cases.

POMs are a unique class of metal–oxygen cluster species usually in nano-sizes ranging from ~5 Å to 40 Å, with versatile composition, scale, structure, charge distribution, and redox potential [10]. They are promising candidates to construct advanced catalytic membranes [11,12], adsorptive membranes [7], and forward osmosis membranes [5], etc. POMs can be integrated into the matrix of the membrane by blending [13] or onto the membrane surface by plasma treatment [14], grafting polymerization [15], and sol-gel coating [16]. When the immobilization of POMs is limited or at the nanoscale, their characterization becomes difficult. Currently, when POMs are immobilized onto/into the membrane surface through the sol-gel method, a deeper understanding of the correlation between the addition of different components (for example, POMs, coupling reagent, sol-gel precursors, and other additives) and membrane structures is deficient. Demand for an effective characterization method of a POMs-functionalized membrane has become urgent. 

Electrochemical impedance spectroscopy (EIS), as an economic and non-invasive characterization technique, can be potentially utilized to study the membrane structures [9,17,18], membrane fouling [19,20], and internal concentration polarization (ICP) [21]. The electrochemical relaxations are related to the intrinsic properties of the studied systems; thus, it can reveal structural features such as structural layers, pore structures, etc., through suitable equivalent circuit modeling. For example, EIS has been successfully used to characterize the polyamide layer of desalination membranes [22,23], epoxy coatings [24], homogeneous membranes [25,26], and biological membranes [27]. Compared with other traditional structural characterization methods, EIS can provide deeper and more considerable information of membrane structures. For instance, Luo and co-workers used EIS to probe the effect of the graphene integrated into the polyvinylidene fluoride (PVDF) membrane matrix on the thermal stability, illuminating that the introduction of graphene improved the membrane stability and suppressed thermal expansion of the pore structures [9]. In addition, EIS also successfully demonstrated that montmorillonite, as a typical two-dimensional (2D) material, adjusted the pore structures of ethyl cellulose membranes, resulting in two different kinds of pores in the composite membranes [28]. Although several studies have been reported to interpret the impedance spectra for different membrane systems, cursory attention should be given to the more complex cases. According to the literature, EIS could be applied to analyze the electrochemical activity and the charged mass transport phenomena of POMs’ layer-by-layer films [29]. Therefore, the current investigation of POMs-modified membranes by EIS has the potential to expand its application and benefit the understanding of other membrane systems.

In this study, a Co mono-substituted Keggin-type [PW_11_O_39_Co(H_2_O)]^4−^ (PWCo), was used as a model POM to fabricate the POMs modified membranes. It has proved as a potent catalyst in various reactions such as the removal of pesticides and dyes [30,31], electrocatalytic oxidation of methanol [32], catalytic oxidation of alcohols [33], etc. [34]. Therefore, the exploitation of transition-metal-substituted POMs-functionalized membranes would promisingly expand their applications as catalytic or electrocatalytic membranes. We designed and fabricated POMs-functionalized membranes with two different routes involving a sol-gel method. The influence of the additives in the sol-forming process on the membrane structures was investigated. The correlation between different fabrication routes and the membrane properties were probed by EIS and discussed based on the equivalent circuit. The POMs distribution and membrane properties were analyzed by EIS as well as other conventional characterization techniques.

## 2. Materials and Methods

### 2.1. Materials and Chemicals

All materials used to make alumina/PVDF membrane substrates included Poly(vinylidene fluoride) (PVDF, Kynar 761) from Arkema (Beijing, China), 1-methyl-2-pyrrolidone (NMP) from Sinopharm Chemical (Shanghai, China), and aluminum sec-butoxide (ASB) from Macklin (Shanghai, China). (3-aminopropyl)trimethoxysilane (APTMS) purchased from Macklin, and nitric acid and iso-propyl alcohol (IPA) from Sinopharm Chemical were also used in the modification process. K_4_[PW_11_O_39_Co(H_2_O)] (PWCo) was synthesized according to the literature [35,36]. All reagents used to fabricate the membrane substrates and PWCo are of analytical reagent (AR) grade and used without further purification.

### 2.2. Membrane Fabrication

**Figure 1:**
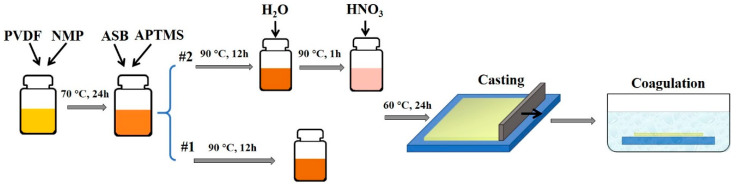
The fabrication process of membrane substrates.

Two kinds of alumina/PVDF flat sheet membranes were fabricated for comparison in subsequent experiments. They were produced via the phase inversion method combined with the sol-gel method. Two bottles of homogenous dope solutions were prepared by the PVDF powder, NMP, under stirring at 70 °C for 24 h. ASB as the alumina precursor and APTMS as an additive were added to both solutions and stirred at 90 °C for 12 h. After that, deionized water was added into one of them (recorded as #2) stirring at 90 °C for 1 h and then nitric acid was added into it stirring at 60 °C for 24 h (PVDF/ASB/APTMS/HNO_3_/H_2_O/NMP at 20:2:1:0.069:0.76:89 weight ratio). The other one which was not added with deionized water and nitric acid was recorded as #1 (PVDF/ASB/APTMS/NMP at 20:2:1:89 weight ratio). The final dope solutions were both cooled down and degassed at room temperature and casted into film with a casting knife whose gate height was 400 μm. The casted film was then immersed into water at room temperature immediately to form a membrane. The resultant membranes (Original#1 and Original#2) were stored in a water bath for at least 3 days and dried in air before use. The preparation process was illustrated in Figure 1. Compared with Original#1, H_2_O and HNO_3_ were added in the dope solution for Original#2. The introduction of H_2_O may promote the hydrolysis rate of the precursors while increasing the instability of the dope system. HNO_3_ was used as a catalyst in the sol-gel process.

### 2.3. Membrane Modification

Before the POMs modification process, the alumina/PVDF membranes were aminated by APTMS. Firstly, both alumina/PVDF original membranes (Original#1 and Original#2) were immersed into the APTMS (2.0 wt%) in IPA/H_2_O (1:1 by weight) solution at 70 °C for 6 h, denoted as APTMS-M#1 and APTMS-M#2. Then, the membranes were rinsed by deionized (DI) water and then immersed into a 4.0 wt% PWCo aqueous solution at 60 °C for 24 h. The resultant membranes (POM-M#1 and POM-M#2) were washed using DI water and dried at room temperature.

### 2.4. Membrane Characterization

The membrane structure and morphologies were characterized with the field emission scanning electron microscope (FESEM, JSM-7600F, JEOL, Tokyo, Japan) operated at a voltage of 5 kV. Energy-dispersive X-ray spectroscopy (EDX) coupled with the FESEM, operated at 10 kV, was utilized to analyze the elemental composition of the membranes. Attenuated total reflectance Fourier transform infrared spectroscopy spectra (ATR-FTIR, IR Presitige-21 FTIR, Shimadzu, Kyoto, Japan) was used to analyze the chemical bonds of the membrane surfaces. The static water contact angle of membranes was measured using a goniometer SL2006 (Kino Tech Corp, Shanghai, China).

### 2.5. EIS Analysis

**Figure 2:**
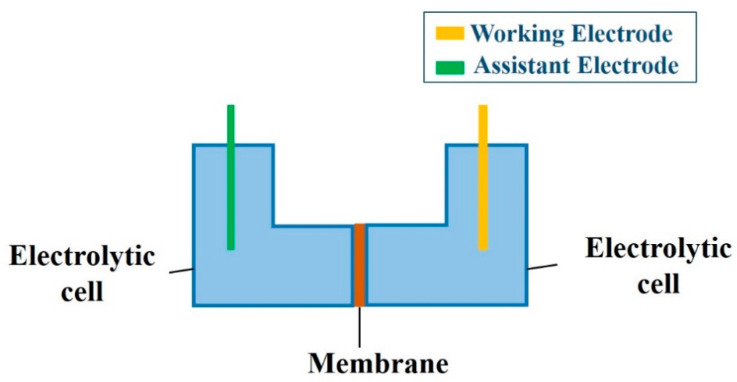
The experimental setup for electrochemical impedance spectroscopy (EIS) measurement.

EIS measurements were performed in 0.01 mol/L KCl aqueous solution at room temperature using a CS310H impedance analyzer electrochemical workstation (Corrtest, Wuhan, China) [28]. The schematic of the EIS device is demonstrated in Figure 2. A membrane with an effective area of 1.77 cm^2^ was fixed between two electrolytic cells. Two platinum electrodes (1 cm^2^ each) were installed in the two electrolytic cells and maintained at the same interval and depth [37]. The impedance spectra were monitored in the frequency range from 0.1 to 10^6^ Hz. The amplitude of the input sinusoidal voltage was 5 mV. The impedance spectra of the as-prepared membrane substrate and modified membranes were tested in 0.01 mol/L aqueous KCl.

### 2.6. EIS Theory and Equivalent Circuit Model

The principle of EIS is based on a sinusoidal potential input applied over a range of frequencies, as shown in Equation (1), and the resulting sinusoidal current across the sample system was measured, as expressed as Equation (2) [38]:

(1) $$E(t)=E_{0}\sin(\omega t)$$

(2) $$I(t)=I_{0}\sin(\omega t+\varphi )$$

where $E_{0}$ and $I_{0}$ represent the maximum of voltage and current, and ω and φ denote the angular frequency and the phase shift, respectively.

The impedance of the membrane system, which is defined as Z(ω)=E(t)/I(t), is expressed as

(3) $$Z=Z^{\prime }+jZ^{\prime \prime }$$

where $Z^{\prime }$ and $Z^{\prime \prime }$ represent the real and imaginary part of the impedance, respectively. The imaginary constant j is defined by $j^{2}=-1$.

Generally, EIS data is illustrated by a Nyquist plot, which maps $Z^{\prime \prime }$ as a function of $Z^{\prime }$, which can reflect interfacial properties between the membrane and solution. The bode plots in the form of |Z| versus frequency or phase angle versus frequency were usually supplemented to provide a direct vision of the magnitude |Z| which equals to $E_{0}/I_{0}$ and phase angle φ in response of frequency. The impedance signal is classically modelled by an equivalent circuit and the equivalent circuit could consist of lumped elements such as resistance, capacitance, and inductance, as well as frequency-dependent elements such as Warburg resistance and constant phase element (CPE).

Typically, a Nyquist plot is modeled by a widely used parallel resistance–capacitance circuit when it is a semicircle with center on the *x*-axis. However, the observed plot deformed occasionally, in which the arc of a circle was shown with the center located below the *x*-axis. In this case, a parallel resistor and CPE circuit is more suitable. The center of the semicircle is located at $(1-n)\times 90^{{}^{\circ}}$. Then, the CPE’s impedance is given as below:

(4) $$Z_{CPE}(\omega )=q^{-1}{\left(i\omega \right)}^{-n}$$

where q represents the factor of proportionality and n stands for the CPE exponent that characterizes the phase shift. For integral values of n (n=1,0,−1), the CPE represents C, R, and L, respectively [39]. Through establishing a suitable equivalent circuit, the membrane physicochemical properties could be associated. For instance, R and C represent the ability to transfer ions and the ability to store electric charges respectively, and CPE could be related with the surface roughness, varying thickness or composition, nonuniform current distribution, and a distribution of reaction rates according to different situations [39].

## 3. Results and Discussion

### 3.1. Membrane Physical and Chemical Properties

**Figure 3:**
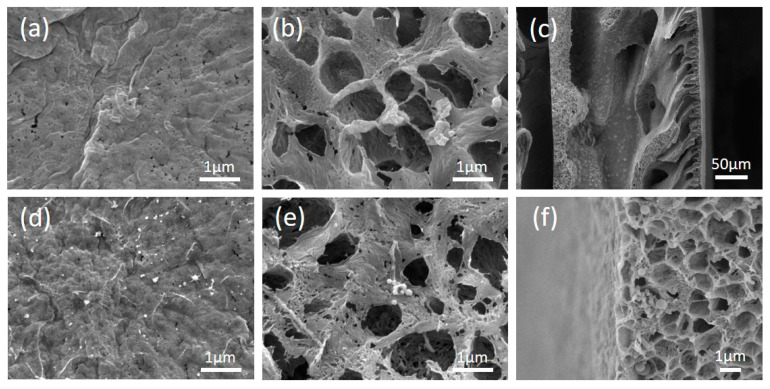
Top view, bottom view, and cross-sectional view of the Original#1 and the modified polyoxometalates (POMs-M#1) membrane by field emission scanning electron microscope (FESEM): (**a**–**c**) for the Original#1 and (**d**–**f**) for the POMs-M#1, respectively.

**Figure 4:**
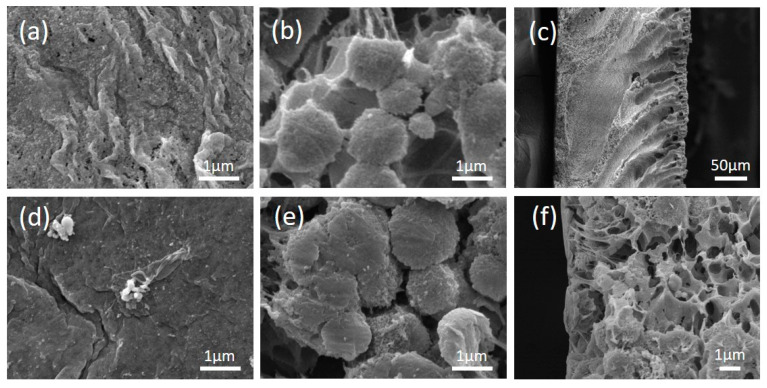
Top view, bottom view, and cross-sectional view of the Original#2 and the modified POMs-M#2 membrane by FESEM: (**a**–**c**) for images of the Original#2 and (**d**–**f**) for the modified POMs-M#2, respectively.

**Figure 5:**
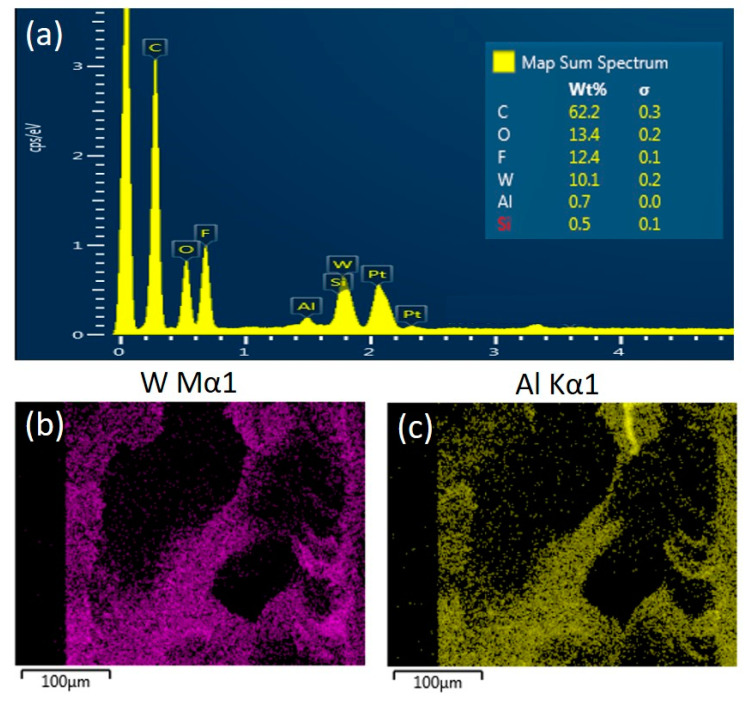
(**a**) Energy-dispersive X-ray spectroscopy (EDX) spectrum of the modified membranes (POMs-M) with inset of atomic contents of main elements and EDX images of (**b**) W and (**c**) Al elements of the cross-section of POMs-M membrane.

Morphologies of the alumina/PVDF composite membranes prepared in two routes (Original#1 and Original#2) and the POMs-modified membranes (POMs-M#1 and POMs-M#2) were analyzed using FESEM and EDX. The results are shown in Figure 3 and Figure 4. The disparity of the original membranes prepared in two routes is the morphology of the bottom layer. It showed that large pores with diameters around 1 μm occupied the bottom layer of Original#1. In contrast, the bottom layer of Original#2 is full of microbeads with diameters around 1 μm. Compared with porous bottom layers, the surfaces of both original membranes were much denser and consisted of pores around 100 nm. As shown in Figure 3c and Figure 4c, a finger-like structure was formed near the surface layer while a sponge-like structure was developed beneath the macro-voids near the bottom layer. As shown in Figure 3d–f and Figure 4d–f, after the two-step modification of POMs for both membranes, limited amounts of nanospheres of POMs can be observed on the membrane surface, bottom, and matrix near the bottom layer. Compared with the original membranes, the pore size of the POMs-M was slightly reduced, evidenced by the images of the surface and bottom layers. EDX analysis (Figure 5) confirmed the successful incorporation of POMs within the membranes. A homogenous distribution of Al and W elements can be observed in Figure 5b,c, demonstrating that alumina and POMs were evenly distributed in amorphous morphologies.

**Figure 6:**
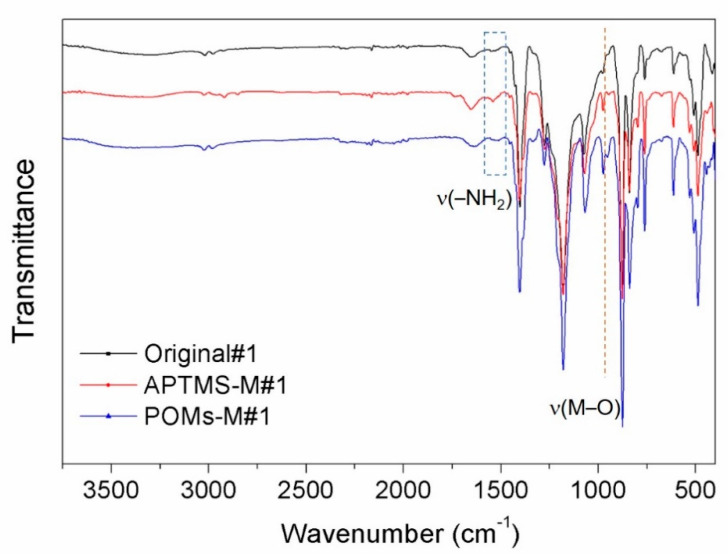
Fourier transform infrared (FTIR) spectra of the original alumina/polyvinylidene fluoride (PVDF) membrane (Orginal#1), the (3-aminopropyl)trimethoxysilane (APTMS)-treated membrane (APTMS-M#1), and the Co mono-substituted Keggin-type [PW_11_O_39_Co(H_2_O)]^4−^ (PWCo)-modified membrane (POMs-M#1).

**Figure 7:**
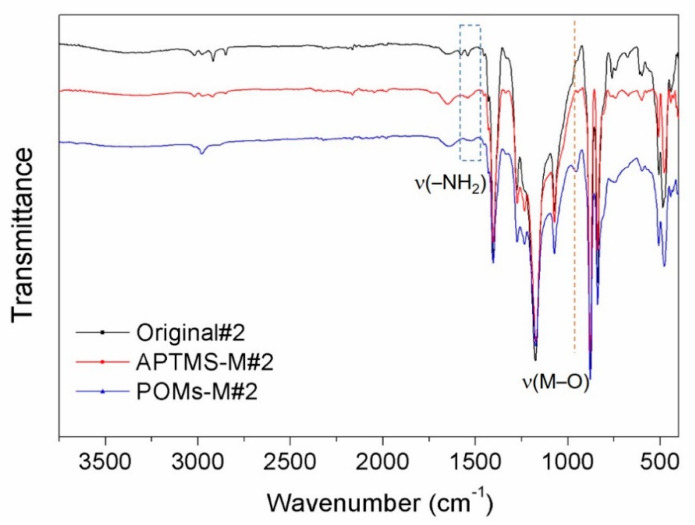
FTIR spectra of the original alumina/PVDF membrane (Orginal#2), the APTMS-treated membrane (APTMS-M#2), and the PWCo-modified membrane (POMs-M#2).

**Figure 8:**
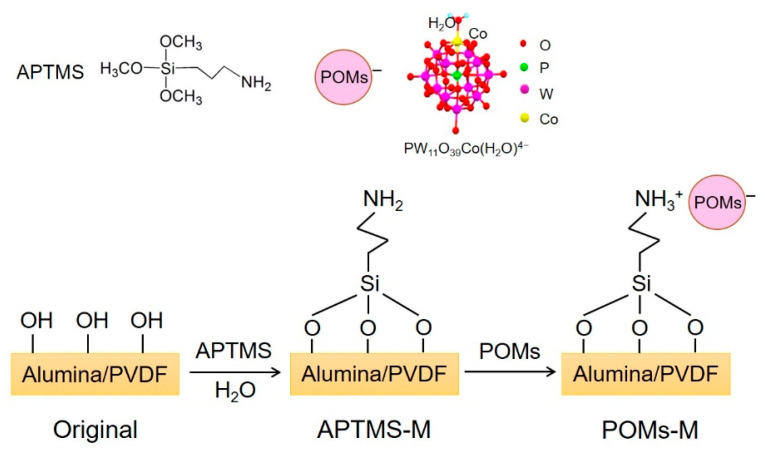
Proposed reaction route for POMs-modified membranes (POMs-M).

The membranes prepared in two routes were further examined by ATR-FTIR and the results are shown in Figure 6 and Figure 7, respectively. For Original#1 and Original#2, after APTMS modification, the peak of –NH_2_, stretching at ~1541 cm^−1^, increased, and revealed a successful incorporation of APTMS on the membrane, while this peak diminished after introducing PWCo (marked by dashed blue box), which was due to the linkage of the –NH_2_ group [40] with the PWCo group through electrostatic interaction. After the incorporation of PWCo, a strong peak emerged at 953 cm^−1^ (marked by the dashed orange lines) which can be the characteristic vibration of the M‒O (M = W or Co) groups of the PWCo Keggin structure. The reaction mechanism is then proposed in Figure 8. Through APTMS treatment, the hydroxyl groups of the original membranes would react with APTMS combined with the hydrolysis and polycondensation of APTMS, resulting in membrane surfaces rich in amine groups. Then, POMs were incorporated onto the membrane through electrostatic interaction under a slightly acidic environment.

The static water contact angle and the pure water permeation (PWP) of the prepared membranes are listed in Table 1. Contact angles of Original#1 and Original#2 are similar. The modification of APTMS would not make much difference on the wettability. After the POMs’ incorporation, the resultant contact angles for both membranes decreased, demonstrating that the membrane surfaces became much more hydrophilic, which is consistent with the hydrophilic nature of POMs. On the other hand, the PWP of POMs-M#1 and POMs-M#2 decreased a little bit compared with the original membranes. 

### 3.2. EIS Testing

**Figure 9:**
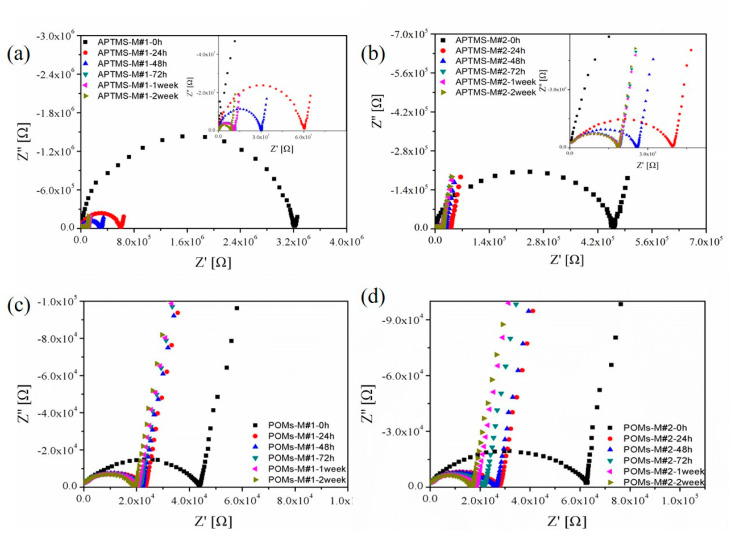
Nyquist plots of EIS analysis in terms of immersion time in 0.01 mol/L KCl solution: (**a**) APTMS-M#1, (**b**) APTMS-M#2, (**c**) POMs-M#1, and (**d**) POMs-M#2.

**Figure 10:**
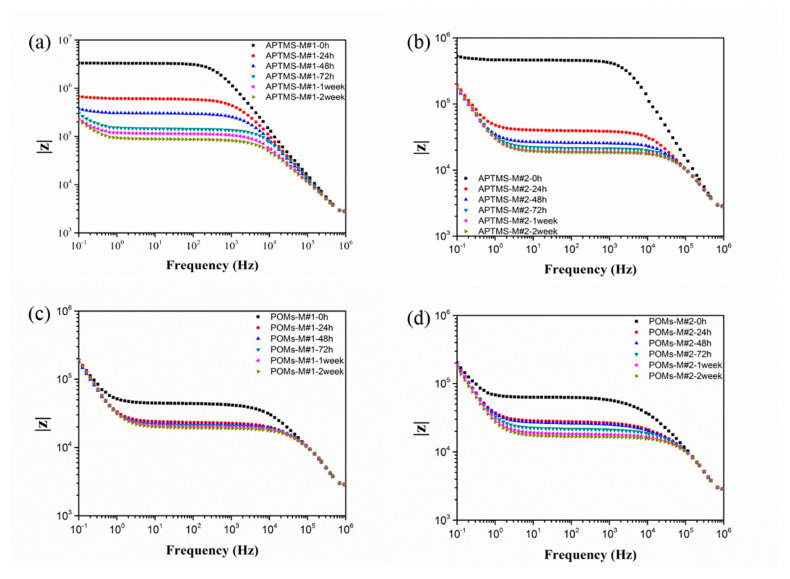
Bode plots of EIS analysis in terms of immersion time in 0.01 mol/L KCl solution: (**a**) APTMS-M#1, (**b**) APTMS-M#2, (**c**) POMs-M#1, and (**d**) POMs-M#2.

**Figure 11:**
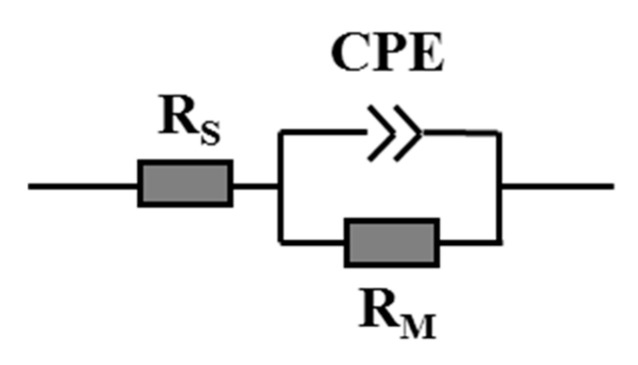
The equivalent circuit of the system.

To investigate the membrane pore structures of the modified membranes, the EIS analysis was conducted. The nature of the polymeric matrix plays a crucial role in the selection of EIS testing parameters. In this study, PVDF, as a chemically stable polymer, was selected as the polymer matrix. Due to its hydrophobic nature, the concentration of the electrolytes should be adjusted to 0.01 mol/L so as to achieve a desirable response signal, while the capacitance characteristic of the electrolytes in the equivalent circuit can be ignored. The Nyquist plots and Bode plots of APTMS-M#1 and APTMS-M#2 membranes immersed in KCl solution for different times are shown in Figure 9 and Figure 10. The Nyquist plot, in which each data point corresponds to a measured frequency, is the most common way to represent the EIS data. Each Nyquist plot in Figure 8 is composed of a depressed semicircle and a tilted spike. The depressed semicircle reflects the membrane/electrolyte interaction, which is highly related with the membrane physicochemical properties, while the tilted spike results from the influence of electrodes and electrolytes. Typically, the larger the semicircle gets, the higher the impedance of the membrane, showing the harder the electrolytes penetrate into the membrane. As shown in Figure 9a,b, the impedance of the APTMS-M#1 and APTMS-M#2 dramatically decreased after a 24 h immersion time and then decreased slightly and remained nearly stable after 1 week. The best fitted equivalent circuit of the system applied in this study consists of series associations of two sections (only impedance arc was studied): resistance R_S_ for the solution between the membrane and the electrode and parallel association of resistance R_M_ for the charge transfer through the membrane and a CPE for the polymer bulk (see Figure 11) [41]. It is worth noticing that CPE fits better than an ideal capacitor for the single time model of experimental data. Table 2 gives a summary of the fitting values using this model. The errors for the calculated R_M_ and CPE-T were less than 1% and 5% respectively, suggesting that the selected equivalent circuit model was desirable. Compared with the 3.16 × 10^6^ Ω resistance for APTMS-M#1 at an immersion time of 0 h, APTMS-M#2 is 4.57 × 10^5^ Ω. This may be related to the greater difficulty of the KCl aqueous solution in filling the pores of the more hydrophobic APTMS-M#1 matrix. Then, the resistance of membranes dramatically decreased and stabilized after 1 week. The final resistance of APTMS-M#1 was 8.80 × 10^4^ Ω, while APTMS-M#2 was 1.92 × 10^4^ Ω, indicating that APTMS-M#2 was slightly more porous than APTMS-M#1. On the other hand, the resistance of POMs-M#1 and POMs-M#2 at 0 h was much smaller than APTMS-M membranes, which may be due to the more hydrophilic nature of the POMs-M membranes, as confirmed by contact angle results. Compared with the 4.37 × 10^4^ Ω resistance for POMs-M#1 at an immersion time of 0 h, POMs-M#2 is 6.47 × 10^4^ Ω. The slightly higher resistance of POMs-M#2 was also compatible with the higher contact angle of POMs-M#2 than POMs-M#1. It implied that the membrane wettability influences the EIS results at the early stage. Finally, the resistance of POMs-M#1 reached 1.99 × 10^4^ Ω, while POMs-M#2 reached 1.71 × 10^4^ Ω. As shown in Table 2, the CPE-T and n are CPE parameters, where n is an adjustable parameter without any real physical basis and its value is generally less than 1 (when n=1, it appears as an ideal capacitor). CPE-T is used in place of a capacitor to show a double layer formation of membrane surface. The similar resistance of APTMS-M#2 and POMs-M#2 after 2-week immersion indicates that the incorporation of POMs through electrostatic attraction would not result in thick deposition. The depressed semicircle which is fitted with a CPE instead of capacitor should be explained by the membrane surface heterogeneity due to surface roughness, which is consistent with the SEM images. It would not influence the pores’ structures of the membranes while introducing hydrophilicity, which is beneficial for anti-fouling.

It can be clearly seen that there was only one semicircle for each Nyquist plot and only one platform region in the Bode plots for all the membranes, suggesting a single time constant in the impedance spectrum. Therefore, though the two-step modification was applied, the membrane model still consists of two compartments: the membrane matrix and the empty volume of the pores. The introduction of APTMS and POMs would not induce the formation of any new kind of pores in the membrane, while the surface roughness and composition were changed. 

## 4. Conclusions

POMs-functionalized PVDF ultrafiltration membranes were fabricated in two routes involving the sol-gel method at different membrane preparation stages. Several conventional characterization techniques were performed to differentiate the membranes. The effects of POMs on the membrane properties were probed by EIS and analyzed based on the equivalent circuit. EIS results showed that the amination of the membrane matrix through APTMS treatment and the immobilization of POMs through electrostatic attraction did not generate new pore structures into the membrane, while the membrane surface roughness and composition were changed. The incorporation of POMs enhanced the membrane hydrophilicity without deteriorating the membrane pore structure and sacrificing the water permeation. It was proven that the wetting degree of the membrane relating to its hydrophilic nature had a crucial influence on the impedance measurements at the early stage. After associating EIS analysis with the evolution of immersion time, the hydrophilic property of the membranes along with their pore structures could be concluded. Therefore, EIS can be potentially applied for probing the structure evolution of different nano-materials-incorporated membranes.

## Figures and Tables

**Table 1:** Comparison of the POMs-M with APTMS-M membrane in terms of the contact angle and pure water permeation (PWP).

Sample Code	Original#1	Original#2	APTMS-M#1	APTMS-M#2	POMs-M#1	POMs-M#2
Pure water flux (L/m2 h bar)	71.0	58.1	92.4	60.1	72.3	57.0
Contact angle (°)	77.2	79.7	85.0	77.2	54.0	64.1

**Table 2:** The fitted values for (**a**) APTMS-M#1, (**b**) APTMS-M#2, (**c**) POMs-M#1, and (**d**) POMs-M#2.

(a)			
APTMS-M#1	R_M_ (Ω)	CPE-T (F)	n
0 h	3.16 × 10^6^	1.85 × 10^−10^	0.95
24 h	6.04 × 10^5^	5.80 × 10^−10^	0.87
48 h	2.99 × 10^5^	8.85 × 10^−10^	0.85
72 h	1.41 × 10^5^	6.10 × 10^−10^	0.86
1 week	1.14 × 10^5^	1.66 × 10^−9^	0.81
2 weeks	8.80 × 10^4^	2.76 × 10^−9^	0.77
**(b)**			
**APTMS-M#2**	**R_M_ (Ω)**	**CPE-T (F)**	n
0 h	4.57 × 10^5^	2.26 × 10^−10^	0.94
24 h	4.07 × 10^4^	3.54 × 10^−9^	0.75
48 h	2.68 × 10^4^	3.49 × 10^−9^	0.77
72 h	2.21 × 10^4^	1.62 × 10^−10^	0.80
1 week	1.95 × 10^4^	4.72 × 10^−9^	0.81
2 weeks	1.92 × 10^4^	1.26 × 10^−9^	0.81
**(c)**			
**POMs-M#1**	**R_M_ (Ω)**	**CPE-T (F)**	n
0 h	4.37 × 10^4^	7.26 × 10^−9^	0.70
24 h	2.42 × 10^4^	4.77 × 10^−9^	0.72
48 h	2.28 × 10^4^	3.07 × 10^−9^	0.75
72 h	2.19 × 10^4^	2.29 × 10^−9^	0.77
1 week	2.11 × 10^4^	2.21 × 10^−9^	0.77
2 weeks	1.99 × 10^4^	2.00 × 10^−9^	0.78
**(d)**			
**POMs-M#2**	**R_M_ (Ω)**	**CPE-T (F)**	n
0 h	6.47 × 10^4^	7.62 × 10^−9^	0.69
24 h	2.89 × 10^4^	7.45 × 10^−9^	0.69
48 h	2.72 × 10^4^	6.77 × 10^−9^	0.69
72 h	2.21 × 10^4^	2.81 × 10^−9^	0.75
1 week	1.89 × 10^4^	1.61 × 10^−9^	0.79
2 weeks	1.71 × 10^4^	1.11 × 10^−9^	0.83
